# Concordance of deregulated mechanisms unveiled in underpowered experiments: PTBP1 knockdown case study

**DOI:** 10.1186/1755-8794-7-S1-S1

**Published:** 2014-05-08

**Authors:** Vincent Gardeux, Ahmet D Arslan, Ikbel Achour, Tsui-Ting Ho, William T Beck, Yves A Lussier

**Affiliations:** 1 Institute for Translational Health Informatics, U. of Illinois at Chicago, IL, USA; 2 Department of Medicine, University of Illinois at Chicago, Chicago, IL, USA; 3 Department of Informatics, School of Engineering, EISTI, Cergy, France; 4 Department of Biopharmaceutical Science, College of Pharmacy, U. of Illinois at Chicago, IL, USA; 5 Robert H. Lurie Comprehensive Cancer Center, Northwestern U., Chicago, IL, USA; 6 Cancer Institute, University of Mississippi Medical Center, Jackson, MI, USA; 7 Department of Bioengineering, University of Illinois at Chicago, Chicago, IL, USA; 8 Computation Inst. & Inst. For Genomics & Systems Biol, Argonne National Lab. & U. of Chicago, IL, USA; 9 Institute for Personalized Respiratory Medicine, U. of Illinois at Chicago, IL, USA; 10 University of Illinois Cancer Center, Chicago, IL, USA; 11 Cancer Ctr, BIO5 Inst., Clinical & Translational Science Inst., U. of Arizona, AZ, USA

## Abstract

**Background:**

Genome-wide transcriptome profiling generated by microarray and RNA-Seq often provides deregulated genes or pathways applicable only to larger cohort. On the other hand, individualized interpretation of transcriptomes is increasely pursued to improve diagnosis, prognosis, and patient treatment processes. Yet, robust and accurate methods based on a single paired-sample remain an unmet challenge.

**Methods:**

"N-of-1-*pathways*" translates gene expression data profiles into mechanism-level profiles on single pairs of samples (one p-value per geneset). It relies on three principles: i) statistical universe is a single paired sample, which serves as its own control; ii) statistics can be derived from multiple gene expression measures that share common biological mechanisms assimilated to genesets; iii) semantic similarity metric takes into account inter-mechanisms' relationships to better assess commonality and differences, within and cross study-samples (e.g. patients, cell-lines, tissues, etc.), which helps the interpretation of the underpinning biology.

**Results:**

In the context of underpowered experiments, N-of-1-*pathways *predictions perform better or comparable to those of GSEA and Differentially Expressed Genes enrichment (DEG enrichment), within-and cross-datasets. N-of-1-pathways uncovered concordant PTBP1-dependent mechanisms across datasets (Odds-Ratios≥13, p-values≤1 × 10^−5^), such as RNA splicing and cell cycle. In addition, it unveils tissue-specific mechanisms of alternatively transcribed PTBP1-dependent genesets. Furthermore, we demonstrate that GSEA and DEG Enrichment preclude accurate analysis on single paired samples.

**Conclusions:**

N-of-1-*pathways *enables robust and biologically relevant mechanism-level classifiers with small cohorts and one single paired samples that surpasses conventional methods. Further, it identifies unique sample/ patient mechanisms, a requirement for precision medicine.

## Background

The emergence of precision medicine ushered in a groundbreaking era in medicine with the opportunity to incorporate individual molecular data into patient care. The variability of individual patients at the molecular level leads to the requirement of individual mechanistic classifiers for accurate prognosis and drug response. However, this individual based-approach requires specific robust statistics, in order to unveil deregulated mechanisms at the level of the single patient, tissue, or cell lines paired samples. Gene expression profile analysis commonly requires a large sample size to achieve sufficient statistical power to uncover deregulated genes or pathways. Yet, such analysis highlights common mechanisms extrapolated to larger population, and overlooks the differences between samples to detect specific individual response to therapy or tissue specific-dependent mechanisms. Therefore, methods are required to empower mechanism-level analysis on a single pairs of samples (tumor vs. matched control, primary tumor vs. metastases, before vs. after treatment samples, etc.). The advent of the increased dynamic range and accuracy of RNA-Sequencing over expression arrays [1,2] provides a new opportunity for studying single subject transcriptomes [3]. N-of-1 clinical trials (or single-subject design) measure patient disease progression or treatment efficacy over time. While molecular biomarker discovery in N-of-1 studies may appear unfeasible, investigation may be headed towards mechanisms and pathways analysis. Indeed, mechanisms-classifiers were shown to outperform gene-level classifiers in addition to reproducible results and advanced understanding of the underpinning biology [4,5].

The proposed method, N-of-1-*pathways*, is able to uncover deregulated pathways at the single patient level, and highlight both individuality and commonality of patient trait or tissue specific associated-pathways [6]. Up to our knowledge, it is the first method that offers the opportunity to leverage individual molecular data for improved diagnosis, prognosis, and patient treatment.

N-of-1-*pathways *relies on three main concepts, which balance statistics, biological modules and information theory: i) a single paired sample is considered the "entire statistical universe", and its genes are the "statistical population" under study (within sample statistic); ii) expressions of multiple genes are combined into genesets as a proxy for biological modules or "pathway" functions; iii) p-values generated for each pathway-associated geneset are sample specific. Hence, in order to conduct cross-studies analyses, semantic similarity metric has been used to reduce the dimensionality of the resulting pathways. Information theory similarity score takes into account inter-mechanisms' relationships, and allows for an unbiased assessment of similarity of pathways conveying the same biologic signal within-sample, cross-samples and across predictions. An unbiased metric of relatedness is crucial as curated hierarchies of classifications and ontologies are arbitrary and inaccurate in assessing relations between genesets. We finally assess common and patient- or sample-specific deregulated mechanisms found by N-of-1-*pathways*, GSEA and DEG enrichment across studies. Taken together, this new method offers opportunity to enhance the underpinning biology across cell/tissue types and between human and animal models.

We conducted these studies to unveil deregulated mechanisms in the context of the alternative splicing factor protein PTBP1 knockdown (Polypyrimidine tract-binding protein 1). PTBP1 was previously reported as a key player in alternative splicing of many genes associated to lineage-specific cell differentiation [7] or tumor genesis [8,9], such as cell cycle. We previously demonstrated that PTBP1 depletion inhibits tumor growth, colony formation and invasiveness *in vitro *in ovarian tumor cells [8,9]. Transcriptome analyses of PTBP1-depleted cells uncover deregulated genesets (mechanisms) and therefore, offer potential therapeutic target discovery. We used one previously reported single paired RNA-Seq sample as well as our new datasets derived from breast and ovarian cancer cell lines, and PTBP1-depleted and matched control samples. We hypothesized that deregulated mechanisms identified in individual samples enable pooled analyses for both "shared pathways" as well as individual results. Further, we compared the "pooled" results with those obtained by conventional geneset enrichment analyses (i) within each dataset when possible (consistency) and (ii) across datasets (validation).

## Methods

***Dataset description*. **Three transcriptome datasets pertaining to PTBP1-depleted cell lines and matched controls were used: Datasets I, II and III. Descriptions are summarized in Table 1 and details of their respective experimental design are described in the first section of the **Results**.

**Table 1:** Transcriptome datasets

Description		Dataset I	Dataset II	Dataset III
**Cell line**		Neuronal cell line (CAD)	Breast cancer cell line (MDA-MB231)	Ovarian cancer cell line (A2780)

**Samples: PTPB1-KD (controls)**		1(1)	4(8)	4(8)

**References**	Authors	Yap K *at al*.	Gardeux V *et al*.	Gardeux V *et al*.
	Source	Genes & Dev.	-	-
	Date	Downloaded 01-2013	2013	2013
	GEO ID	GSE37933	GSE52493	GSE52493

**Expression measurements**	Type	RNA-Seq	Microarray GeneChip	Microarray GeneChip
	Platform	Genome Analyzer IIx Illumina	Prime View Human Gene Expression Array	Prime View Human Gene Expression Array

**Measured transcripts or probes**		27389	**49395**	**49395**

**Deregulated transcripts or genes**		707	**720**	**469**

***RNA-Seq dataset and preprocessing (Dataset I)***. The RNA-Seq dataset (Table 1) pertains to transcriptomes of PTBP1-depleted mouse neuroblastoma cell line CAD (Cath. A-Differentiated; a variant of CNS catecholaminergic cell line, Cath. A) and matched controls. The read counts are normalized by RPKM (Reads Per Kilobase of transcript per Million mapped reads). All measurements were log2 transformed. If several alternative transcripts referring to the same HGNC gene name were present, only the one with maximum expression was considered for further analysis. To minimally transform or bias the data, we processed all the experiments without filtering genes with low expression. The entire GEO control and PTBP1-KD RPKM data (1+1 samples) were used for N-of-1-*pathways *analysis, while the list of 1.5-fold deregulated genes between control and PTBP1-KD samples was provided and further enriched with the Fisher's Exact Test.

***Cell lines, culture conditions (Dataset II and III)*. **The epithelial human breast cancer cell line MDA-MB231 (ER-/ PR-/ HER2-) were obtained from the American Type Culture Collection (Manassas, VA). The epithelial human ovarian tumor cell line A2780 was received as a generous gift from Dr. Thomas C. Hamilton (Fox Chase Cancer Center, Philadelphia, PA) Cancer Center, Philadelphia, PA). Cells were grown in DMEM supplemented with 10% fetal bovine serum (FBS), 2mM L-glutamine in a humid environment at 37°C, with 5% CO_2_. Both cell lines were free of *Mycoplasma *species and were maintained for no longer than 10 weeks in culture after recovery from frozen stocks. *Mycoplasma *levels were checked periodically using the MycoAlert^® ^Mycoplasma Detection Kit (Lonza Inc., Allendale, NJ). The authenticity of cell lines was assessed by the ATCC carrying out short tandem repeat (STR) analysis (Verified STR Profiling Service, ATCC^® ^135-XV). Additionally, we compared A2780 to the original STR profile collected by the European Collection of Cell Culture (Catalogue number 93112519).

**Figure 1:**
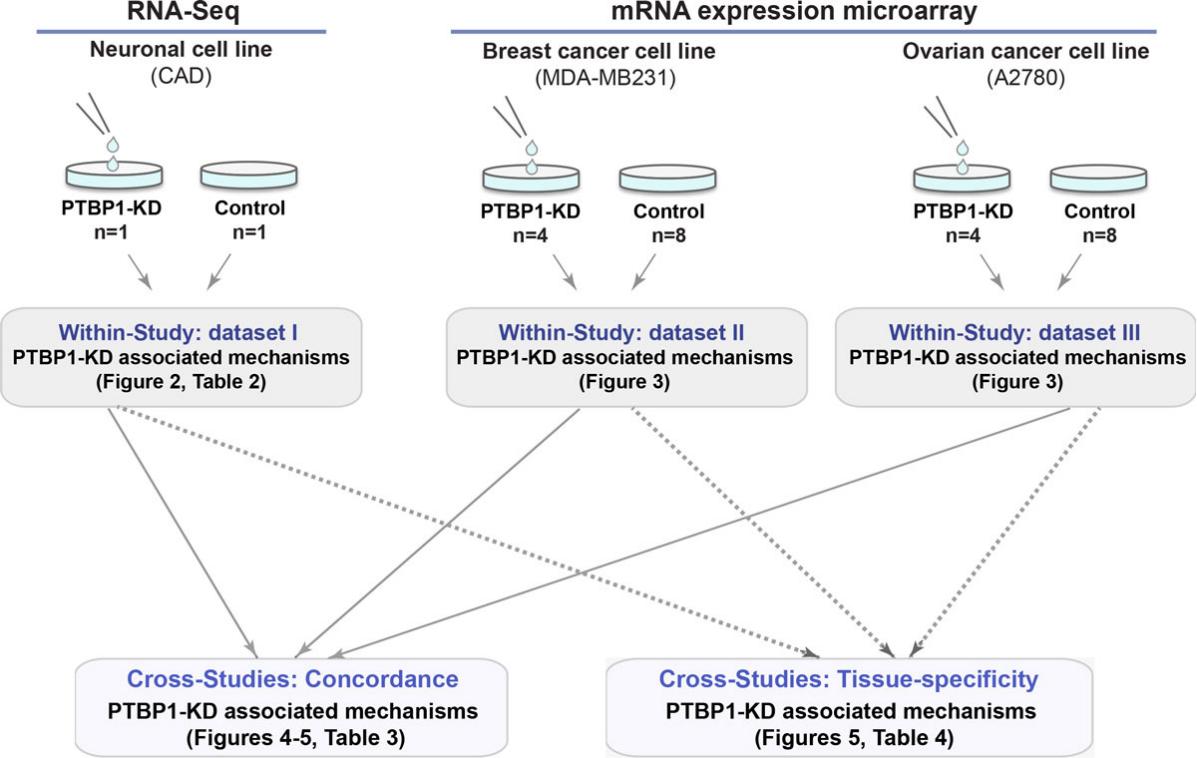
**Experimental design**.

***Doxycycline-inducible knockdown of PTBP1 regulated by small hairpin RNA *(*shRNA; Datasets II and III)***. In order to analyze the effect of PTBP1 depletion, two consecutive viral transductions were performed in both MDA-MB231 and A2780 cell lines. Cells were plated on 24-well plate (10-20 × 10^4 ^cells/well), maintained in culture for 16 hours, and then medium containing LV-tTR/KRAB-Red lentiviral particles was added. Following 16 h of incubation, cells were transduced a second time by LVTHM/PTBshRNA or LV-THM/LUCshRNA lentiviral particles. Clones expressing both red and green fluorescent protein (dsRED and GFP respectively) were selected and expanded. Following 16 h of incubation, cells were washed and split in two subcultures, one without doxycycline (PTBP1/-DOX; **Control in **Figure 1) and the other with Doxycycline (DOX) at a final concentration of 1 µg/ml (PTBP1/+DOX; **PTBP1-KD in **Figure 1). Doxycycline was prepared according to the manufacturer's recommendations (Sigma-Aldrich, St. Louis, MO). Five days later, cells were analyzed by fluorescence microscopy, and PTBP1 gene expression was assessed using PCR and Western Blotting (data not shown). The cells that were transduced by LV-LUCshRNA express PTBP1 regardless of the presence of DOX (LUCshRNA/+DOX). Constructs and lentivirus preparation were performed as previously described [9].

***Microarray Analysis (Dataset I and II)*. **For each of the cell lines, MDA-MB231 and A2780, total RNAs were extracted from four biological replicates of PTBP1-depleted cells, PTBP1-KD (4 × PTBP1/+DOX) and eight biological replicates control cells (4 × PTBP1/-DOX and 4 × LUCshRNA/+DOX) by Direct-zol RNA kit (Zymo Research, Irvine, CA) (Figure 1). All paired samples consist of PTBP1-depleted cells (PTBP1KD) and matched control cells. Qualities of RNA were assessed based on the RNA quality indicator (RQI ≥ 8) using Experion Automated Electrophoresis System (Bio-Rad, Hercules, CA). Gene expression microarray measurements were performed using the GeneChip PrimeView Human Gene Expression Array that contains 49,395 probes and measures 36,000 transcripts and variants per sample. Labeling and hybridization were performed following Affymetrix protocols. The raw data were normalized according to the Robust Multiple-array Average (RMA) technique [10], using Affymetrix Power Tools (APT) [11]. The complete set of raw and normalized data is available for download on the GEO database (**GSE52493**; Table 1).

***Gene Ontology annotations of Biological Processes (GO-BP) ***[12,13]. We aggregated genes into pathway-level mechanisms using Gene Ontology Biological Process, GO-BP. Hierarchical GO terms were retrieved using the *org.Hs.eg.db *package [14] (Homo Sapiens) and the *org.Mm.eg.db *package [15] (Mus Musculus) of *Bioconductor *[16], available for R statistical software [17]. We used the *org.Hs.egGO2ALLEGS *database (downloaded on 03/15/2013), which contains a list of genes annotated to each GO term (*geneset*) along with all of its child nodes according to the hierarchical ontology structure. The genesets were filtered so that only those sized between 15 and 500 were kept in the studies. These GO annotations were used for three types of GO prioritization analyses: GSEA, DEG Enrichment and N-of-1-*pathways *analysis (described below in **Methods**).

***Kyoto Encyclopedia of Genes and Genomes (KEGG) ***[18,19]**. **We aggregated genes into pathway-level mechanisms using Kyoto Encyclopedia of Genes and Genomes, KEGG. KEGG pathways were retrieved using the *org.Hs.eg.db *package [14] (Homo Sapiens) and the *org.Mm.eg.db *package [15] (Mus Musculus) of *Bioconductor *[16], available for R statistical software [17]. We used the *org.Hs.egPATH *database (downloaded on 03/15/2013), which contains a list of genes annotated to each KEGG pathway (*geneset*). The genesets were filtered so that only those sized between 15 and 500 were kept in the studies. These KEGG annotations were used for three types of KEGG prioritization analyses: GSEA, DEG Enrichment and N-of-1-*pathways *analysis.

**Figure 2:**
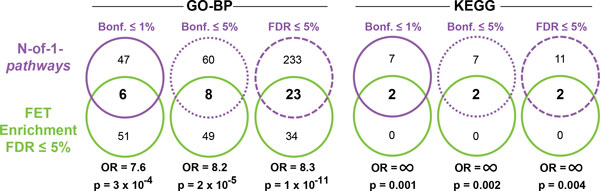
**WITHIN-STUDY, Dataset I concordance of PTBP1-KD associated mechanisms unveiled by N-of-1-*pathways *and FET Enrichment in RNA-Seq neuronal cell line**. The Venn Diagrams correspond to the overlap of deregulated mechanisms, GO-BP terms (left panel) and KEGG pathways (right panel) found between N-of-1-*pathways *(at different cutoff, Bonf. ≤ 1%, Bonf. ≤ 5% and FDR ≤ 5%) and FET Enrichment method (FDR≤5%). The Odds Ratios (OR) and p-values are shown below each Venn Diagram. GSEA is not represented, as it cannot be computed with a single paired sample.

**Figure 3:**
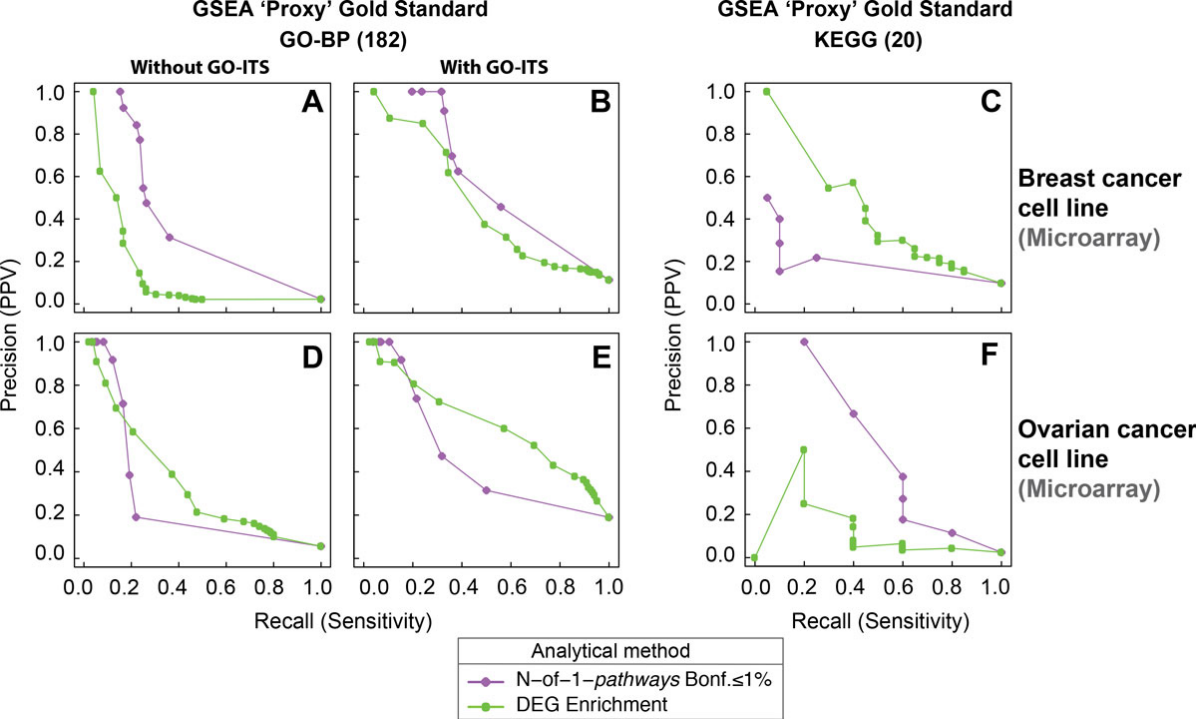
**WITHIN-STUDY concordance of PTBP1-KD associated mechanisms found by N-of-1-*pathways *compared to those found by GSEA and DEG Enrichment, applied to breast and ovarian cancer gene expression microarray profile (Datasets II-III)**. To evaluate the GO-BP and KEGG associated terms of deregulated mechanisms yielded by the N-of-1-*pathways *method in both breast and ovarian cancer internal studies, we compared these mechanisms to those found by DEG Enrichment when GSEA is chosen as the 'Proxy' Gold Standard (Proxy GS, Methods). We then generated precision-recall curves based on the exact GO overlap (Without GO-ITS, panels A, D), related GO terms by Information Theory Similarity overlap (With GO-ITS, panels B, E; GO-ITS ≥ 0.7; Methods), and the exact KEGG overlap (panels C, F).

**Figure 4:**
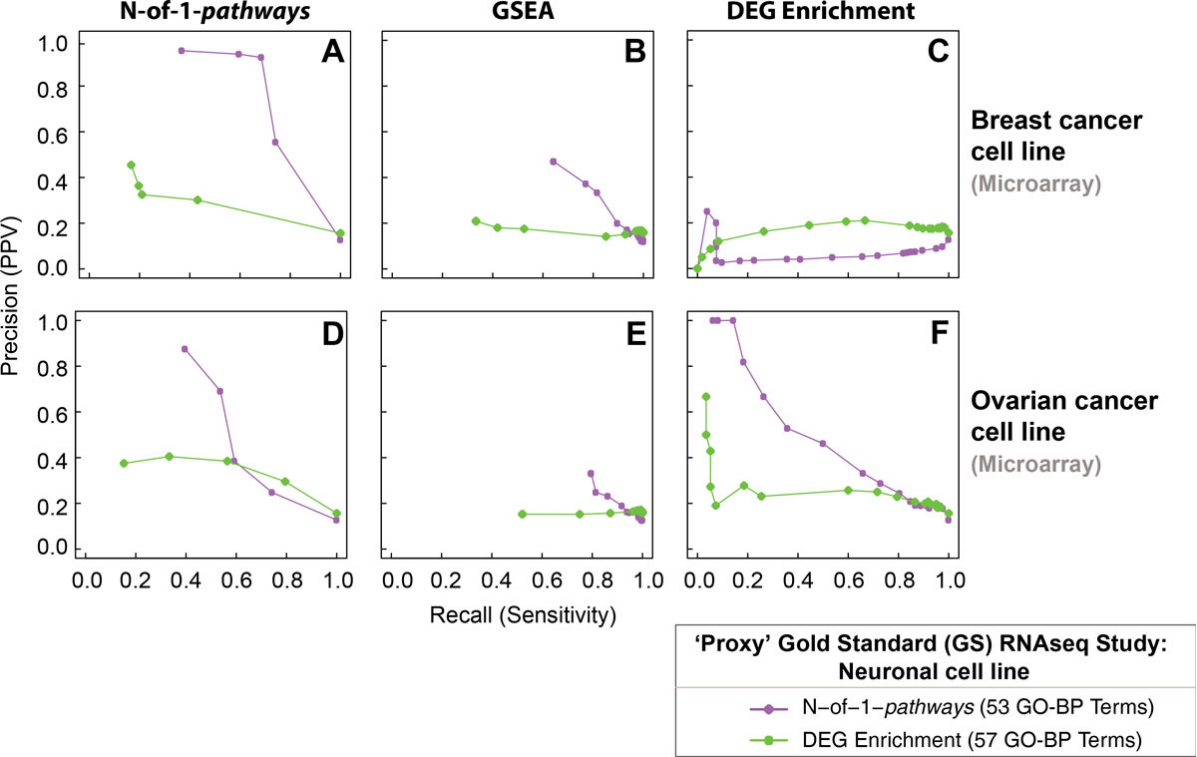
**CROSS-STUDIES concordance of PTBP1-KD associated mechanisms found by N-of-1-*pathways *and conventional methods in breast and ovarian cancer cell lines using neuronal cell line mechanisms as Gold Standard**. We compared mechanisms unveiled by N-of-1-*pathways *and DEG Enrichment in neuronal cell lines to those associated in breast and ovarian cancer cell lines and found by all three methods. We set RNA-Seq neuronal cell related results as a 'Proxy' Gold Standard (Proxy GS; **Methods**) and generated precision-recall curves using GO-BP semantic similarity overlap (GO-ITS ≥ 0.7; **Methods**).

**Figure 5:**
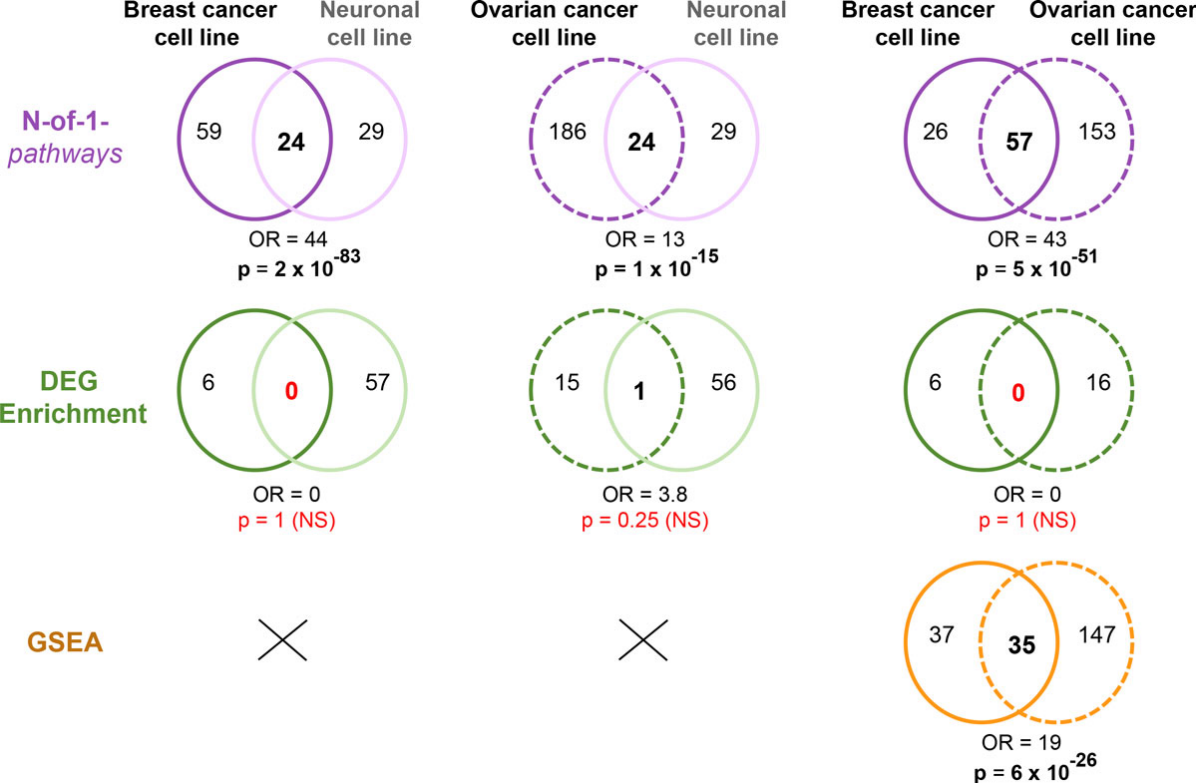
**CROSS-STUDIES accuracy of mechanism identification methods using their default parameters**. Strong overlap performance of N-of-1-*pathways *method. We compared the three different mechanism identification methods (N-of-1-*pathways*) across the three different studies (neuronal, breast and ovarian cancer cell lines). The computed overlaps of PTBP1-KD associated mechanisms are represented by Venn Diagrams. Odds Ratio (OR) and p-values (p) are plotted below the Venn Diagrams to represent the statistical significance of the overlap (**Methods**). The symbol "X" marked in the GSEA results represents not computed analysis, as this method cannot be applied to the single paired sample form the neuronal cell line dataset I.

***N-of-1-pathways method applied to in vitro / in vivo experiments***[6]***(Figures ***2, 3, 4, 5***).*** MECHANISMS PRIORITIZED WITHIN ONE PAIR OF SAMPLES: The N-of-1pathways method was performed on the three datasets (Table 1, **Datasets I, II, III**) independently for each paired sample (PTBP1-KD and control, Figure 1). The first set of the proposed method consists of a non-parametric paired Wilcoxon test (Wilcoxon signed-rank test) performed within each sample on the paired gene expression profiles restricted to a given mechanism. Wilcoxon statistics, W^+ ^and W^−^, provide direction on deregulated genesets as overall "up-regulated" or ''down-regulated'' respectively. Both FDR and Bonferroni (Bonf.) corrections were applied to adjust p-values for multiple comparisons. In each paired sample, only deregulated mechanisms with adjusted p-values with FDR ≤ 5%, Bonf. ≤ 1% or Bonf. ≤ 5% were retained for further analysis. MECHANISMS PRIORITIZED ACROSS MULTIPLE PAIRS OF SAMPLES: For comparison of the N-of-1-*pathways* method with cross-patient enrichment of mechanisms, a second step is required to prioritize the mechanisms otherwise found in individual pairs of samples. Each mechanism has an associated p-value for each paired sample. The p-values were then ranked according to the total number of samples sharing a given mechanism that reached significance at Bonf. ≤ 1% (default suggested cutoff parameter). The prioritized mechanisms were listed from the most commonly to the least observed across samples, yet significant in at least one sample. Adjusted p-values are then transformed into Z-scores for further within- and cross-samples analyses. The N-of-1-*pathways* software is available in R and Java at http://Lussierlab.org/publications/N-of-1-pathways

***Gene Sets Enrichment Analysis (GSEA)*. **Gene set enrichment analysis was conducted on breast and ovarian cancer datasets only (Table 1, **Datasets II, III**). In the case of the neuronal dataset, GSEA was not performed as it is underpowered with a single pair of samples (Table 1, **Dataset I**). The GSEA v2.0.10 software [20] was used with the default parameters except for the permutation parameter selection, which was set to "geneset" instead of "phenotype". Geneset permutation was chosen to achieve enough statistical power for permutation resampling due to the small number of samples.

***Mechanisms enriched from Differentially Expressed Genes (FET and DEG Enrichment; ***Figures 2, 3, 4, 5***) ***Enrichments of GO-BP and KEGG genesets with differentially expressed (DE) genes were conducted in the R statistical software using the Fisher's Exact Test (FET) based on the following contingency table: (DE genes, All Genes) × (In Pathway, Not In Pathway). Adjustment for multiple comparisons was performed using Benjamini and Hochberg method (False Discovery Rate; FDR), and mechanisms with FDR ≤ 5% were considered significantly enriched. Of note, the up-regulated and down-regulated genes were enriched independently. DE genes were directly available for neuronal RNA-Seq study, but only based on fold change cutoff (Table 1, **Dataset I**). We called "FET Enrichment" the enrichment of those deregulated genes to avoid any mixed up with the standard DEG Enrichment. The breast and ovarian cancer DE genes (Table 1, **Datasets II, III**) were calculated in the following way: (i) genes whose average expression differs by at least 2-fold between Control (8 samples) and PTBP1-KD samples (4 samples) were selected for analysis, (ii) then a t-test was applied between the two groups, and p-values were adjusted with Benjamini and Hochberg method (False Discovery Rate; FDR). Only DE genes with FDR ≤ 5% were retained.

***Information Theoretic Similarity (ITS) ****(only applicable for GO-BP mechanisms; *Figures 3 and 4***)*. **In order to further stratify mechanisms in those that are unique to a pair of samples or common to multiple samples, Information-Theory Similarity (ITS) is utilized to formally assess similarity cross sample pairs versus uniqueness to a pair. When applied on samples from an individual patient, this method allows determining mechanism unique to a patient versus those common to many, a step forward in personal therapy from transcriptome data. We calculated the similarity between GOBP terms using Jiang's information theoretic similarity [21] that ranges from 0 (no similarity) to 1 (exact match).

***Within-Study Proxy Gold Standard (***Figure 3***)*. **Mechanisms are statistically prioritized in breast and ovarian cancer datasets by the three above described methods: N-of-1-*pathways*, GSEA and DEG-Enrichment. The accuracy of the N-of-1*pathways *method was compared to one of the conventional methods (eg. DEG Enrichment) while the other serves as a Proxy Gold Standard (GSEA). ***Cross-Studies derived Gold Standards (***Figure 4***)*. **Significant deregulated mechanisms in PTBP1 depleted neuronal cell lines unveiled by N-of-1-*pathways *and DEG Enrichment methods (Table 1, Dataset I) were used as Proxy Gold Standard. For the DEG Enrichment method, the list of DEG was directly provided by the authors and further enriched. These two lists of mechanisms serve as derived Gold Standards to compare their robustness across studies, methods, and underpinning biology (PTBP1 depleted cells; mouse *versus *human, neuronal *versus *cancer cell lines; breast *versus *ovarian cancer cell lines.) ***Precision-Recall curves (***Figures 3, 4***)*. **Using the R statistical software, we computed two types of Precision-Recall curves: (i) within-study (Figure 3) and (ii) cross-studies (Figure 4) of the mechanisms predicted by the N-of-1-*pathways *(Cross-samples; see above), GSEA and DEG Enrichment. *WITHIN-STUDY*: Precision-recall curves of the "internal validation" compare breast and ovarian cancer GO-BP and KEGG associated mechanisms unveiled by the N-of-1-*pathways *with those predicted by DEG Enrichment and GSEA that were used alternatively as "Proxy Gold Standard" (Proxy GS) (Figure 3). *CROSS-STUDIES*: Breast and ovarian cancer GO-BP and KEGG associated mechanisms uncovered by the N-of-1-*pathways*, GSEA and DEG Enrichment were compared to those found in the RNA-Seq neuronal dataset by the Nof-1-*pathways *and DEG-Enrichment (considered as GS) (Figure 4). *STANDARD PRECISION-RECALL CURVE: *The GS list of deregulated mechanisms are fixed (given a particular cutoff) while the precision and recall point of each mechanism identification method is ranked either according to its p-values (GSEA and DEG Enrichment) or the number of samples (N-of-1-*pathways*). The precision and recall values are calculated using different cutoffs of the ranked mechanisms derived from the prediction methods. In this case, a true positive is calculated as an overlap between a prediction and the GS. A true negative corresponds to a mechanism neither predicted nor found in the GS. A false positive is a predicted mechanism not found in the GS while a false negative corresponds to non-predicted GS mechanism.

***Information-Theory Similarity (ITS) in precision-recall curve (only applicable for GO-BP mechanisms): ***for these precision-recall curves, we considered a true positive prediction if the predicted mechanism is similar to a mechanism from the GS (ITS ≥ 0.7). We have previously shown that an ITS score ≥ 0.7 robustly corresponds to highly similar GO terms using different computational biological validations: protein interaction [22,23], human genetics [24], and Genome-Wide Association Studies [25].

***Statistical significance of overlap of two lists of mechanisms (Odds Ratio, OR; and p-value; ***Figures 2,5***)*. **In order to assess the statistical significance of mechanism overlap unveiled by two different methods, we computed the following contingency table: (#Overlapping mechanisms, #Non-overlapping mechanisms in method 1) × (#Non-overlapping mechanisms in method 2, #Remaining mechanisms in mathematical universe). We then computed an odds ratio (OR) and a p-value using the Fisher's Exact Test (FET). The computed p-value obtained with FET is equivalent using a Hypergeometric Test.

## Results

### Overview of the datasets and performed studies

Figure 1 provides an overview of the experimental design. We evaluated the robustness of the N-of-1-*pathways *method in three transcriptome profile datasets: a single paired sample (RNA-Seq from mouse neuronal cell line, Dataset I) and two small sets of paired samples (mRNA expression microarray from human breast and ovarian cancer cell lines, Dataset II and III). Each dataset consists of a number *n *of PTBP1-depleted samples and matched controls. To uncover PTBP1-KD associated mechanisms, we first performed within-study analyses (N-of-1-*pathways*, FET enrichment, GSEA, DEG enrichment) independently for each of the datasets I, II and III (**Methods**, Figures 2, 3**; **Table 2; GSEA and DEG was not applicable to Dataset I). We then quantitatively and qualitatively compared the three analytical methods across the three studies to reveal concordant and tissue/cell specific deregulated mechanisms associated to PTBP1-KD (Figures 4, 5**; **Table 3).

### Within-study (Dataset I). Concordance of PTBP1-KD associated mechanisms unveiled by N-of-1-*pathways *and FET Enrichment in neuronal cell line

We first performed an independent within-study analysis from published RNA-Seq transcriptome profile of PTBP1-depleted neuronal cell lines (**Dataset I; **Table 2; Figure 2). To apply geneset-level enrichment analysis, conventional methods such as GSEA and DEG enrichment require three samples to reach statistical significance. The present dataset I consists of a single sample of PTBP1-KD and a single paired Control (*n *= 2) and therefore GSEA and DEG enrichment are not the methods of choice for such underpowered experiments. We applied our proposed method, N-of-1-*pathways*, which is designed for these experiments while only a Fisher's Exact Test (FET) Enrichment analysis could be performed on genes at a certain fold change level, as differentially expressed genes cannot be calculated with a p-value between two samples. Arguably, FET enrichment is barely applicable with n = 2, since only a fold change of differentially expressed genes between two samples can be measured and considered for further analysis. To our knowledge, no method can compute a p-value at the mRNA level (gene-level) from a single paired sample analysis of RNA-seq based-transcriptome. We then show significant overlap of PTBP1-KD associated mechanisms prioritized by both methods using GO-BP and KEGG genesets (Figure 2; p < 0.05; Odds Ratio: OR>7). The different Odds Ratios (OR) were comparable regardless of the multiple comparison cutoffs of the N-of-1-*pathways *method (Bonf. ≤ 1%, Bonf. ≤ 5%, FDR ≤ 5%). In order to limit the number of false positive results for N-of-1-*pathways *and produce succinct, interpretable, lists of mechanisms, we favored a Bonferroni ≤ 1% in the remainder of this paper while we kept a FDR ≤ 5% for GSEA and DEG Enrichment. Thereafter, we evaluated the biological relevance of such GO-BP associated mechanism overlaps between N-of-1-*pathways *and FET Enrichment (Figure 2 leftmost panel; Table 2). Six GO-BP were found overlapping and ten others were found related based on ITS semantic similarity computed score (ITS ≥ 0.7, Methods). All together, the 16 mechanisms belong to three GO-BP classes: Cell Cycle/DNA Replication, DNA repair and neuronal transmission. Indeed, N-of-1-*pathways *and FET enrichment recapitulate the tissue-specific deregulated mechanisms of synaptic transmission and synaptic vesicle exocytosis that were previously confirmed in biologic assays by the study from which the RNA-Seq dataset I was generated [26]. The authors showed that the depletion of PTBP1 affects alternative splicing and triggers transcriptome changes on a large scale, which increased the propensity of the neuronal CAD cells to undergo neuron-like differentiation. However, unlike N-of-1-*pathways*, FET enrichment did not uncover alternative splicing related mechanisms.

**Table 2:** GO-BP overlap and similarity between N-of-1-*pathway*s and FET Enrichment derived from RNA-Seq transcriptome profile of PTBP1-depleted neuronal cell line.

Curated GO-BP classes	GO-BP Terms	GO-BP overlap	GO-BP ITS ≥ 0.7*	Neuronal specific
Cell cycle and DNA Replication	GO:0006260: DNA Replication	✓		
	GO:0051325: interphase	✓		
	GO:0007067: mitosis		✓	
	GO:0051329: interphase of mitotic cell cycle		✓	
	GO:0010564: regulation of cell cycle process		✓	
	GO:0033261: regulation of S phase		✓	
	GO:0000279: M phase		✓	
	GO:0000087: M phase of mitotic cell cycle		✓	
	GO:0006974: response to DNA Damage Stimulus	✓		

DNA repair	GO:0006281: DNA Repair	✓		
	GO:0006310: DNA recombination	✓		
	GO:0006302: double-strand break repair		✓	

Neuronal transmission	GO:0007268: synaptic transmission	✓		✓

*GO-BP terms with Information Theoretic Similarities (ITS) ≥ 0.7 are considered highly related **(Methods)**.

### Within-study, datasets II and III: concordance of PTBP1-KD associated mechanisms unveiled by N-of-1-*pathways*, DEG Enrichment and GSEA in Breast and Ovarian cancer cell lines

We performed a within-study analysis independently for two different human cancer cell lines (breast and ovarian cancer; Table 1, **Dataset II, III**). Each study consists of four biological replicates of PTBP1-KD and eight matched control (not depleted PTBP1) samples. We applied GSEA, DEG Enrichment and N-of-1-*pathways *methods independently on each dataset I and II and compared their accuracies (Figure 3). The results show that three out of the six N-of-1-*pathways *method predictions were found comparable or better to those of DEG enrichment when GSEA is chosen as a Gold Standard (GS) (Figure 3 Panels A, B and F). Compared to DEG Enrichment, the proposed N-of-1-*pathways *offers the same level of results with the advantage of being applicable to a single paired sample. Additional File 1**- Supp. Figure S1 **shows the results taking DEG Enrichment as the Proxy GS. However, DEG Enrichment did not provide enough statistically significant deregulated pathways to perform an accurate comparison.

### Cross-studies: concordance of PTBP1-KD associated mechanisms unveiled by N-of-1-*pathways*, DEG Enrichment and GSEA across all three datasets

Using either N-of-1-*pathways *or DEG Enrichment as the two alternate Gold Standards (performed in the neuronal cell lines), N-of-1-*pathways *surpasses well-known methodologies in five out of six predictions conducted in ovarian and breast cancer cell lines (Figure 4). Specifically, mechanisms discovered by N-of-1-*pathways *method applied in the RNA-Seq neuronal cell line dataset are highly concordant to those found in breast cancer and ovarian cancer (Figure 4, Panels A and D). Taken together, the precision and recall curves of each method in breast and ovarian cell lines (vertical columns) and the overall accuracies of GSEA (Figure 4, Panels B and E) and DEG Enrichment (Figure 4, Panels C and F) are lower than those of N-of-1-*pathways *(Figure 4, Panels A and D), regardless of the GS used (GS from neuronal study). Of note, N-of-1-*pathways *performs better than GSEA and DEG Enrichment with only one exception (Figure 4, Panel C). Moreover, the concordance of DEG Enrichment fails in breast cancer cell lines (Figure 4, Panel C) as well as in ovarian cancer cell lines (Figure 4, Panel F). Therefore, both consistency and robustness of the mechanisms unveiled by DEG Enrichment across datasets are questionable. In summary, these results highlight the advantage of applying the N-of-1-*pathways *for a single paired sample analysis compared to conventional methods.

To qualitatively assess the biologic relevance of mechanism overlap, we curated the associated and unrelated GO-BP deregulated mechanisms across the three datasets found by the N-of-1-*pathways *method. As shown in Table 3, N-of-1-*pathways *discovered the most common and highly related mechanisms previously reported [8,9,26] as associated to molecular and cellular phenotypes that are triggered by PTBP1 depletion such as GO:0000398, mRNA splicing via spliceosome and GO:00010564, regulation of cell cycle process. We also studied the dissimilar mechanisms between datasets; Additional File 2**-Supp. Tables S1-S3 **displays sets of GO-BP classes underlying tissue-specific mechanisms affected by PTBP1 depletion. ***For the neuronal cell line (***Additional File 2***- Supp. Table S1)*, **10 GO-BP are clustered in 3 GO-BP classes, such as RNA localization/transport including GO:0051028, mRNA transport and Nucleic acid transport including GO:0015931 nucleobase-containing compound transport. ***For the breast cancer cell line (***Additional File 2***- Supp. Table S2)***, 10 GO-BP are clustered in 4 GO-BP classes such as cytokine production class including GO:0001816, regulation of cytokine production and immune response class including GO: cellular response to type I interferon. ***For the ovarian cancer cell line (***Additional File 2***-Supp. Table S3)***, 117 GO-BP are clustered in 10 curated class such as, hormone secretion/process class including GO:0046883, regulation of hormone secretion; organ/tissue development class including GO:0030855, epithelial cell differentiation and GO:001655 urogenital system development; pathway signaling class including GO:0007179, transforming growth factor beta receptor signaling pathway. Although some deregulated mechanisms could help to decipher tissue-specificity of PTBP1 role in alternative transcription, further investigations are required to understand their underpinning biology.

**Table 3:** Concordance of regulated mechanisms by PTBP1 across three cell lines (neuronal, breast cancer and ovarian cell lines) discovered by N-of-1-*pathways*.

GO-BP Classes	GO-BP Overlap		GO-BP ITS ≥ 0.7	
RNA splicing/RNA processing	GO:0008380	RNA splicing	GO:0000398	mRNA splicing, via spliceosome
	GO:0006397	mRNA processing	GO:0000375	RNA splicing, via transesterification reactions
			GO:0000377	RNA splicing, via transesterification reactions with bulged adenosine as nucleophile
			GO:0016071	mRNA metabolic process
			GO:0034470	ncRNA processing
			GO:0006364	rRNA processing

Cell cycle/cell division	GO:0007067	mitosis	GO:0000279	M phase
	GO:0051301	cell division	GO:0000216	M/G1 transition of mitotic cell cycle
	GO:0000280	nuclear division	GO:0000082	G1/S transition of mitotic cell cycle
	GO:0000087	M phase of mitotic cell cycle	GO:0000086	G2/M transition of mitotic cell cycle
	GO:0051325	interphase	GO:0000236	mitotic prometaphase
	GO:0051329	interphase of mitotic cell cycle	GO:0051320	S phase
	GO:0006260	DNA replication	GO:0000084	S phase of mitotic cell cycle
	GO:0007059	chromosome segregation	GO:0000819	sister chromatid segregation
	GO:0071156	regulation of cell cycle arrest	GO:0006261	DNA-dependent DNA replication
	GO:0010564	regulation of cell cycle process	GO:0000070	mitotic sister chromatid segregation
	GO:0000075	cell cycle checkpoint	GO:0007093	mitotic cell cycle checkpoint
	GO:0000226	microtubule cytoskeleton organization	GO:0045786	negative regulation of cell cycle
	GO:0007017	microtubule-based process	GO:0010948	negative regulation of cell cycle process
	GO:0048285	organelle fission	GO:0007346	regulation of mitotic cell cycle
			GO:0051439	regulation of ubiquitin-protein ligase activity involved in mitotic cell cycle
			GO:0031023	microtuble organizing center organization
			GO:0051327	M phase of meitic cell cycle
			GO:0007051	spindle organization
			GO:0007126	meiosis
			GO:0051321	meiotic cell cycle

Chromatin modifications/ remodeling			GO:0016568	chromatin modification
			GO:0006325	chromatin organization
			GO:0016569	covalent chromatin modification
			GO:0016570	histone modification
			GO:0051052	regulation of DNA metabolic process

DNA repair	GO:0006310	DNA recombination	GO:0006974	response to DNA damage stimulus
	GO:0006281	DNA repair	GO:0006302	double-strand break repair

Neuronal process			GO:0031644	regulation of neurological system process
			GO:0007268	synaptic transmission
			GO:0050804	regulation of synaptic transmission
			GO:0051969	regulation of transmission of nerve impulse

Others			GO:0007600	sensory perception

### Cross-studies: tissue-specific and concordance of mechanisms regulated by PTBP1 unveiled by N-of-1-*pathways*, DEG Enrichment and GSEA across all three studies

We further evaluate the reproducibility of each mechanism based-method and their robustness across studies using Venn Diagrams and Odds Ratios (OR; Figure 5). Since PTBP1 has a major role in alternative splicing mechanism, its depletion in all three type of cells (Neuronal, breast and ovarian) provides an advantage to determine the accuracy of PTBP1 common and tissue-specific deregulated mechanisms. Interestingly, unlike N-of-1-*pathways*, the lack of reproducibility of DEG Enrichment results across studies prevents the recovery of significant overlap. While GSEA provided good overlap (OR≥19) between breast and cancer cell line datasets, it failed to provide an overlap between these studies and the neurological dataset, as it cannot be applied to a single paired sample studies (eg. dataset I). In contrast, N-of-1-*pathways *can be applied robustly in each case scenario achieving overall the best performance with high OR ≥ 13 at significant p-values (p ≤ 1 × 10^−15^) far surpassing those of GSEA and of DEG Enrichment in every combination of dataset. Taken together, N-of-1-pathways is able to provides both PTBP1 common and tissue-specific deregulated mechanisms independently of the sample size do datasets.

## Discussion

**Future studies. **In the context of paired samples, the validation studies results are so favorable for N-of-1-*pathways *that we are planning large-scale studies (synthetic and real datasets) systematically comparing N-of-1-*pathways *to multiple conventional geneset enrichment methods. Further, we are investigating the scalability of N-of-1-*pathways *in genome-wide measurements other than the transcriptome (e.g. methylation) to reveal mechanisms of resistance to therapy.

**Limitations**. At the biological level, the large extent of shared mechanisms between RNA-Seq (Dataset I) and mRNA expression microarrays (Datasets I and II) attests the sheer ability of N-of-1-*pathways *to be applied across platforms. However, unlike the neuronal RNA-Seq dataset, the two newly generated datasets submitted to GEO were conducted using microarrays without exon-specificity measures, preventing the identification of alternative transcripts. Therefore, shared mechanisms such as cell cycle, RNA processing, and splicing need further experimental investigations to reveal the underpinning biology of PTBP1 in regards to alternative splicing. At the computational level, simulation across samples is required to establish the dynamic range of precision and recall of N-of-1-*pathways *as compared to geneset enrichment studies. The methodology should be extended to single samples rather than paired samples using a different unpaired rank statistic and reference samples from GEO (underway). Moreover, as a large number of GO-BP may be found deregulated within two paired samples, GO-ITS scores could be further automated in order to reduce the dimensionality and facilitate the interpretation of the results.

## Conclusions

In the present study, we established a novel methodology, N-of-1-*pathways*, empowering mechanism-based analysis using as few as two samples. N-of-1-*pathways *relies on three principles. First, the statistical universe is a single patient or a set of paired samples. Second, mechanisms unveiled within paired samples can be measured from genesets. Indeed, multiple measures for each mechanism can be obtained and a statistic can be derived. Third, the "naive" exact overlap of mechanism's coded terms is not sufficient to assess commonality or differences between patients or between pairs of samples. A formal similarity metric is required to take into account the hierarchy and/or the shared genes among mechanisms' genesets. To extrapolate general population-level conclusions, popular comparative study analyses require achieving sufficient statistical power based on a large sample size. Here, statistical power is attainable despite a small sample size: a single patient (or cell line, or tissue, etc.) with as few as 2 samples. Yet, population-based generalizations can be conducted by merging significant individual results together. Thus, we compared the results of N-of-1-*pathways *with two conventional methods: GSEA and DEG Enrichment, which are well-known pathway-level techniques applied to large sample sets. So far the results show that our method surpasses previous mechanism-discovery methods even if it was originally designed to identify the deregulated mechanisms at the single patient-or paired sample-level. Importantly, novel translational bioinformatics methods provide advanced understanding of the dynamic range of PTBP1 role in regulating alternative transcript expression of genes associated to proliferation, invasiveness, drug-resistance, etc. Such methods offer the opportunity to serve as proof-of-concept, paving the way to potential therapeutic agents to be investigated, such as small molecules and biologics inhibiting aberrant PTBP1 expression as in the case of ovarian cancer and glioma. Further, the N-of-1-*pathways *method is likely to be scaled up to a new type of mechanism, such as "chromoplexy". Recently, this unveiled phenomenon showed the interdependency and biologic modularity of somatic mutations from which oncogenicity emerges [27,28] rather than the old paradigm of one single point mutation to trigger an oncogenic phenotype.

Taken together, the increased accuracy for population-based study and the sub-group stratification empowered by this computational biology method prepares the path to leverage individual molecular data for profoundly improved mechanistic classifiers of prognosis and chemotherapeutic response. Recent DNA sequencing results support the massive somatic mutation differences in individual patient cancers [29,30]. Therefore, it is important to further develop patient-specific interpretations and high throughput experiments that support off-label medication repositioning for individualized precision therapy.

## List of abbreviations

Bonf., Bonferroni correction for multiple comparisons; DE Genes, Differentially Expressed Genes; DEG enrichment, Differentially Expressed Genes Enrichment; DOX, Doxycycline; FDR, False Discovery Rate; FET, Fisher's Exact Test; GO, Gene Ontology; GO-BP, Gene Ontology - Biological Processes; GS, Gold Standard; GSEA, Gene Sets Enrichment Analysis; KEGG, Kyoto Encyclopedia of Genes and Genomes; ITS (or GO-ITS), Information Theoretic Similarity; OR, Odds Ratio; PTBP1, Polypyrimidine Tract-Binding Protein 1; PTBP1-KD, PTBP1 Knockdown; RPKM, Reads Per Kilobase of transcript per Million mapped reads.

## Competing interests

The authors declare that they have no competing interests.

## Authors' contributions

Conceived the computational & biologic experiments: YAL, WTB, VG, IA, AAD, TTH; conducted the computational biology analyses: VG, IA, YAL; knowledge base: WTB, IA, YAL; analyzed the results: VG, IA, YAL; wrote and revised the manuscript: VG, IA, WTB, YAL.

## Supplementary Material

Additional file 1 Supplementary Figure S1 - WITHIN-STUDY concordance of PTBP1-KD associated mechanisms found by N-of-1-*pathways *compared to those found by GSEA and DEG Enrichment, applied to breast and ovarian cancer gene expression microarray profile (Datasets II-III). To evaluate the GO-BP and KEGG associated terms of deregulated mechanisms yielded by the N-of-1-*pathways *method in both breast and ovarian cancer internal studies, we compared these mechanisms to those found by GSEA when DEG is chosen as the 'Proxy' Gold Standard (Proxy GS, Methods). We then generated precision-recall curves based on the exact GO overlap (Without GO-ITS, panels A, D), related GO terms by Information Theory Similarity overlap (With GO-ITS, panels B, E; GO-ITS ≥ 0.7; Methods), and the exact KEGG overlap (panels C, F). Click here for file

Additional file 2 **Supplementary Tables S1, S2 and S3**. This file contains the three supplementary tables in numbering order. Those tables list the mechanisms regulated by PTBP1 in neuronal, breast and ovarian cell lines, respectively. Click here for file

## Declarations

Publication for this article and the study were funded in part by NIH grants UL1TR000050, K22LM008308 and the University of Illinois Cancer Center as well as funded in part by NIH/NCI CA138762 (to WTBeck) and by the University of Illinois at Chicago. This work was supported in part by the UA Cancer Center grant (NCI P30CA023074), the UA BIO5 Institute, and the UA Clinical & Translational Science Institute of the University of Arizona.

This article has been published as part of *BMC Medical Genomics *Volume 7 Supplement 1, 2014: Selected articles from the 3rd Translational Bioinformatics Conference (TBC/ISCB-Asia 2013). The full contents of the supplement are available online at http://www.biomedcentral.com/bmcmedgenomics/supplements/7/S1.
