# Supplementary material for: Concordance of deregulated mechanisms unveiled in underpowered experiments: PTBP1 knockdown case study
Source: BMC Med Genomics. 2014 May 8;7(Suppl 1):S1. doi: 10.1186/1755-8794-7-S1-S1 (PMC4101571; doi:10.1186/1755-8794-7-S1-S1)

Additional file_1

Supp. Figure S1. WITHIN-STUDY concordance of PTBP1-KD associated mechanisms found by N-of-1-*pathways* compared to those found by GSEA and DEG Enrichment, applied to breast and ovarian cancer gene expression microarray profile (Datasets II-III). To evaluate the GO-BP and KEGG associated terms of deregulated mechanisms yielded by the N-of-1-*pathways* method in both breast and ovarian cancer internal studies, we compared these mechanisms to those found by GSEA when DEG is chosen as the 'Proxy' Gold Standard (Proxy GS, Methods). We then generated precision-recall curves based on the exact GO overlap (Without GO-ITS, panels A, D), related GO terms by Information Theory Similarity overlap (With GO-ITS, panels B, E; GO-ITS ≥ 0.7; Methods), and the exact KEGG overlap (panels C, F).

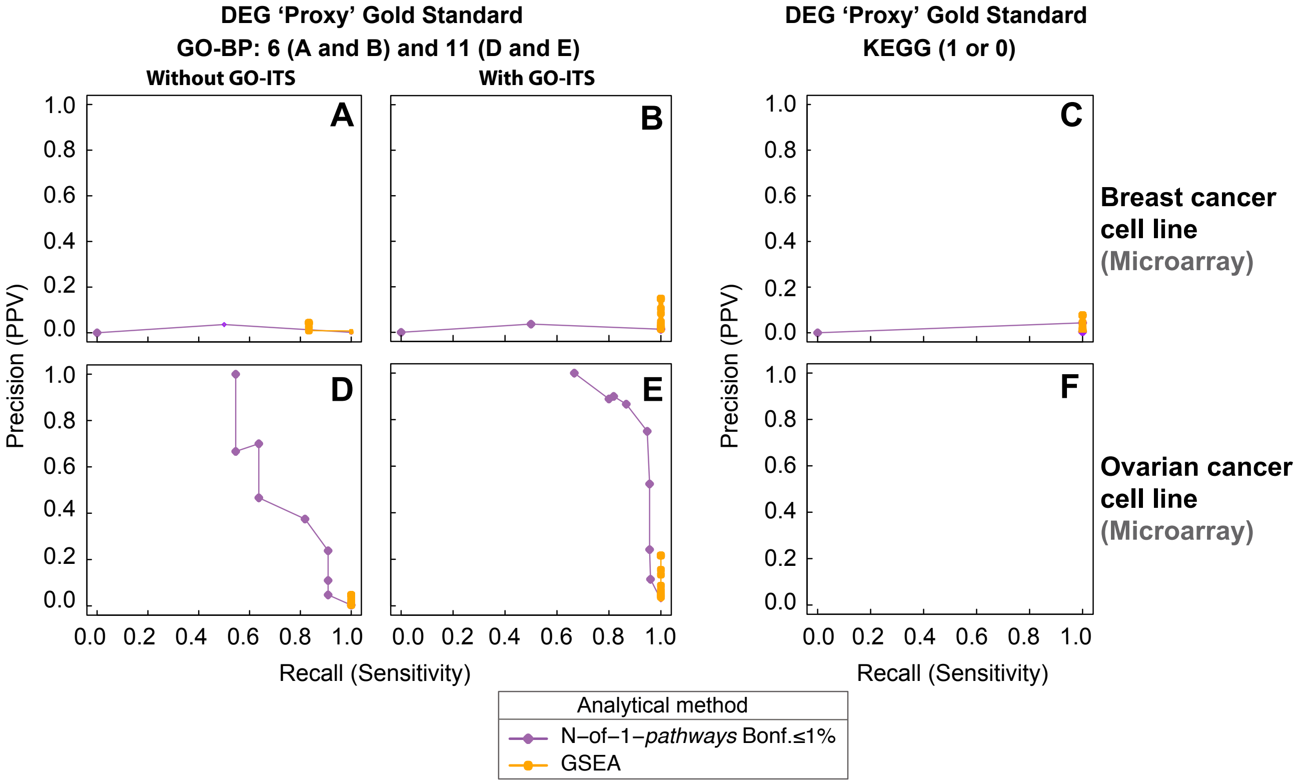

Supplement: Additional file 1 — Supplementary Figure S1 - WITHIN-STUDY concordance of PTBP1-KD associated mechanisms found by N-of-1-pathways compared to those found by GSEA and DEG Enrichment, applied to breast and ovarian cancer gene expression microarray profile (Datasets II-III). To evaluate the GO-BP and KEGG associated terms of deregulated mechanisms yielded by the N-of-1-pathways method in both breast and ovarian cancer internal studies, we compared these mechanisms to those found by GSEA when DEG is chosen as the 'Proxy' Gold Standard (Proxy GS, Methods). We then generated precision-recall curves based on the exact GO overlap (Without GO-ITS, panels A, D), related GO terms by Information Theory Similarity overlap (With GO-ITS, panels B, E; GO-ITS ≥ 0.7; Methods), and the exact KEGG overlap (panels C, F). [file 1755-8794-7-S1-S1-S1.PDF]
